# Cultural Adaptation and Implementation Strategy of a Recovery‐Oriented Mental Health Training Intervention (REFOCUS‐THAIREC) for Healthcare Workers in Thailand: An Experience‐Based Co‐Design

**DOI:** 10.1111/hex.70738

**Published:** 2026-06-22

**Authors:** Natthapon Inta, Annmarie Grealish, Mary Leamy

**Affiliations:** ^1^ Florence Nightingale Faculty of Nursing, Midwifery & Palliative Care King's College London London UK; ^2^ Princess Agrarajakumari Faculty of Nursing Chulabhorn Royal Academy Bangkok Thailand; ^3^ School of Nursing and Midwifery, Health Research Institute University of Limerick Limerick Ireland

**Keywords:** cultural adaptation, educational training, experience‐based co‐design, healthcare professionals, implementation strategy, mental health recovery, recovery‐oriented intervention, REFOCUS, Thailand

## Abstract

**Background:**

Healthcare workers are key in supporting mental health recovery, though the approach is poorly understood in many Asian countries. In Thailand, no recovery‐oriented training currently exists, highlighting the need for a culturally adapted intervention that incorporates locally meaningful concepts and clarifies unfamiliar recovery principles. The present study aimed to culturally adapt an existing recovery training intervention (REFOCUS/REFOCUS‐PULSAR) for use by healthcare workers in Thailand (REFOCUS‐THAIREC).

**Methods:**

Experience‐Based Co‐Design (EBCD) was used to culturally adapt content and implementation strategies of the REFOCUS/REFOCUS‐PULSAR interventions, guided by the ADAPT framework and the Model for Adaptation Design and Impact. Thirty‐one participants were involved one or more of the EBCD stages, including healthcare professionals (HCPs), people with mental illness (PwM), and carers.

**Results:**

Four key adaptations were identified: a catalyst film illustrating service user experiences, a recovery‐oriented care planning component, two case studies illustrating recovery journeys, and a recovery‐oriented language guide. Six implementation strategies were proposed, including experiential learning, digital delivery options, preparation for lived experience trainers, separate training streams for professional and non‐professional groups, practical care planning tools and the use of recovery outcome measures.

**Conclusion:**

REFOCUS‐THAIREC provides a culturally relevant training approach for Thai healthcare workers and a foundation for implementing recovery‐oriented practice in community mental health services.

**Patient or Public Contribution:**

Mental health service users, carers and HCPs were involved as partners in the co‐design process and reviewed research materials, including the Thai Global INSPIRE questionnaire, interview topic guide and training manua.

## 1 Introduction

Personal recovery is grounded in adaptability, resilience and having a sense of control over one's life, enabling individuals with mental illness to achieve wellbeing and satisfaction [1], and ultimately empowering them to live meaningful lives and become valued members of society [2]. Central to achieving this goal are healthcare staff who work closely with people with mental illness (PwM). Although the idea of personal recovery was conceptualised around two decades ago in Western culture [3, 4], a limited understanding of recovery concepts in non‐Western countries, such as Thailand, poses challenges to the implementation of recovery‐oriented care [5].

Training in mental health recovery has become a promising approach to strengthen healthcare workers’ recovery‐oriented practice [6]. Training programmes have been available in the United Kingdom (UK) since 2005, and National Institute for Health and Care Excellence (NICE) guidelines recommend that staff training should focus on recovery principles to ensure that all rehabilitation staff practise in a recovery‐oriented way [7]. Previous research indicates that recovery‐oriented training not only improves staff knowledge, attitudes, skills and competencies [8, 9], but also contributes to service users’ recovery outcomes, including their psychological wellbeing, quality of life and empowerment [10]. However, despite Thailand introducing recovery‐oriented care into its national mental health policy in 2019, and into the operational plan of the Department of Mental Health 2023–2027 [11, 12], no recovery‐oriented trainings have been developed for or implemented within Thai mental health services [13]. Clearly, the development of evidence‐based recovery training and education programmes within Thai healthcare would offer an opportunity to strengthen staff knowledge, attitudes, skills and competencies in order to better promote recovery‐focused practice.

The ADAPT guidance recommends adapting evidence‐based interventions for new contexts as a more efficient and sustainable approach than developing new ones [14]. Frameworks such as the Model for Adaptation Design and Impact (MADI) support this process by systematically identifying adaptation characteristics and their influence on implementation outcomes [15], as demonstrated in adaptations for veterans with post‐traumatic stress disorder [16]. Such approaches enhance contextual relevance and cultural compatibility by engaging stakeholders in adaptation decisions, whilst reducing the risk of conflict with local values [17, 18]. A recent systematic review of international and Thai published and grey literature identified 20 recovery‐oriented training programmes, grouped into seven categories based on their theoretical foundations [13]. Using the Structured Assessment of Feasibility (SAFE) tool [19], the REFOCUS training programme [20] was identified as a promising candidate for adaptation in non‐Western settings. REFOCUS is a team‐level training intervention, co‐delivered by professionals and people with lived experience, and grounded in the CHIME framework of recovery [21]. The REFOCUS intervention is a research‐based programme aimed at enhancing recovery support in mental health services. It focusses upon two main components—recovery‐promoting relationships and working practices. Recovery‐promoting relationships involve coaching skills training for staff, developing a shared understanding of recovery and exploring staff values. Working practices include understanding values and treatment preferences, assessing strengths and supporting goal striving by service users. Developed in the United Kingdom, it is delivered through face‐to‐face training for community mental health workers and has demonstrated effectiveness in two randomised controlled trials [10, 22]. It has also been successfully adapted in other Western contexts, including Australia (REFOCUS‐PULSAR) [10], and most recently, in France (REFOCUS‐RETAFORM) [23].

**Figure 1:**
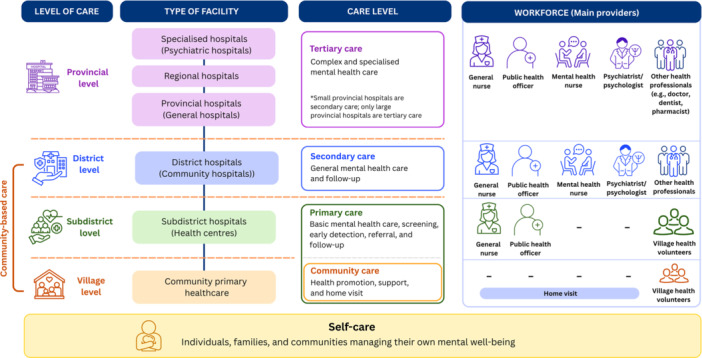
Thai mental health service system and workforce.

Cultural adaptation extends beyond translation or modification, involving thoughtfully reshaping an intervention to align with the values, beliefs and lived experiences of a specific cultural context [24]. Indeed, the success of any intervention depends largely on how well it resonates with the people and systems within that context [25]. In the Thai healthcare context, mental health care is integrated into the public health system [26] (see Figure 1). In rural and community settings, particularly in subdistrict hospitals, mental health care is inevitably delivered by non‐mental health professionals, such as general nurses and public health officers, whose responsibilities span a wide range of activities including mental health care, maternal and child health, vaccination, substance use prevention, infectious disease control and management of non‐communicable diseases [27, 28, 29]. However, even with care being provided by these (non‐mental) healthcare professionals (HCPs), the workforce remains insufficient and caseloads are often disproportionally high. As a result, community‐based physical and mental health care is frequently supported by non‐professional workers, such as village health volunteers (VHVs) [28]. These VHVs receive basic training in health promotion, health education and disease control [30], and have supported health service delivery in rural Thailand for over four decades [31]. Similar to other Asian countries [32, 33], mental health recovery as an individualised and service user‐centred concept remains relatively unfamiliar in Thailand, as reflected in the care planning process, where care plan has traditionally been clinician‐led, with clinicians playing a central role in decision‐making [34]. In Thailand, Nursing care plans are commonly structured around the North American Nursing Diagnosis Association (NANDA) guideline, which is grounded in deficit‐ and illness‐focused approaches that prioritise impairments and depend largely on professional judgement to identify care needs [35]. This approach may reduce opportunities for service users to express their own perspectives and has raised concerns that maintaining control and managing risk can be prioritised over therapeutic support and person‐centred care [36].

Perspectives on mental health in Thailand are deeply rooted in Buddhist teachings, which strongly influence cultural beliefs and traditional values. Buddhism encourages self‐reflection and awareness through the acceptance of one's thoughts and feelings, whilst promoting resilience and emotional regulation as ways of coping with mental distress [37]. These principles are further reinforced by the values of empathy and compassion, which support psychological wellbeing, encourage individuals to find meaning and happiness through caring for others [38], and help facilitate the resolution of interpersonal and intergroup conflicts [39]. Although gratitude is regarded as an important human attribute, in Buddhism it refers to a sense of appreciation and the moral obligation to respond positively to acts of kindness [40]. This concept is closely linked to family relationships, particularly within Thai families, where younger generations are expected to show respect and gratitude towards older family members [41]. Given the strong value placed on family connectedness, family members are often actively involved in providing support and participating in healthcare decision‐making processes [42]. Therefore, family involvement is an essential component of mental health care in Thailand, particularly when supporting mental health recovery. To address the limited knowledge of recovery and the lack of recovery‐oriented training in Thailand, an existing recovery training intervention should be implemented. However, these contextual factors must be carefully considered when culturally adapting the intervention to the Thai context to ensure its relevance and appropriateness.

## 2 The Study

### 2.1 Aims

This study aimed to collaborate with Thai stakeholders to identify priorities for recovery‐oriented care and to co‐design and culturally adapt the REFOCUS/REFOCUS‐PULSAR training intervention for the Thai context (REFOCUS‐THAIREC), as well as to develop contextually appropriate implementation strategies informed by the MADI framework.

### 2.2 Research Questions

1. What priorities for recovery‐oriented care do Thai stakeholders identify?
2. How can these priorities inform the cultural adaptation of the REFOCUS/REFOCUS‐PULSAR training intervention and its implementation strategies?

## 3 Methods

### 3.1 Study Design

In this study, we employed Experience‐Based Co‐design (EBCD) [43, 44] as a collaborative process to systematically adapt the REFOCUS/REFOCUS‐PULSAR intervention for Thailand (see Figure 1). The process was informed by the ADAPT guidance, introduced in 2021 as a framework to support the efficient adaptation of interventions with established evidence bases for use in new cultural or service contexts [14]. ADAPT was appropriate given its emphasis on contextual relevance, stakeholder involvement and preserving core intervention components, aligning with the co‐design approach used in this study.

The study also employed the MADI [15], which offers a structured approach for documenting, planning and evaluating adaptations to interventions or implementation strategies, helping researchers understand why adaptations occur and how they influence implementation/intervention outcomes. The MADI comprises of three domains (adaptation characteristics, mediating or moderating factors and implementation/intervention outcomes) to consider the causal pathway of adaptations and their intended and unintended impacts on implementation and intervention outcomes [15].

**Figure 2:**
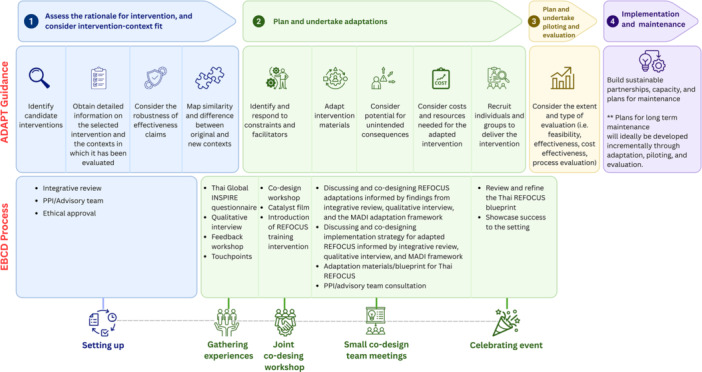
Integrated co‐design‐ADAPT guidance.

This study was built upon findings from our systematic review of recovery‐oriented mental health training interventions [13] and thematic synthesis of stakeholders' recovery experiences [45]. The overview of the key elements of the intervention co‐design process is presented in Figure 2. This study is reported in line with the guidance for reporting intervention development studies in health research (GUIDED) [46].

### 3.2 Participants, Settings and Recruitment

The study was conducted with PwM, carers and HCPs from one district hospital and three subdistrict health promoting hospitals in Chiang Mai, Thailand. Following ethical approval granted by King's College London (Reference number HR/DP‐24/25‐45539) and site approval from Chiang Mai Provincial Public Health Office (Reference number CM0033.010/2721), data collection was started in April 2025 and completed in September 2025.

Purposive sampling was used to recruit 12‐15 HCPs and 12‐15 service users (PwM and carers) [47] through face‐to‐face contact, by the first author (N.I.), who was unfamiliar with the settings to reduce power dynamics, during routine appointments and poster advertisements at a district hospital mental health clinic and three subdistrict hospitals. Adults diagnosed with either mental illness or substance‐related mental illness, who had been receiving community mental health care; adult carers who cared for those PwM; and HCPs involved in primary and/or community mental health care were recruited. Participants received an information sheet outlining the study's aims, procedures, confidentiality safeguards, voluntary participation and provided written informed consent before joining the co‐design workshops.

**Figure 3:**
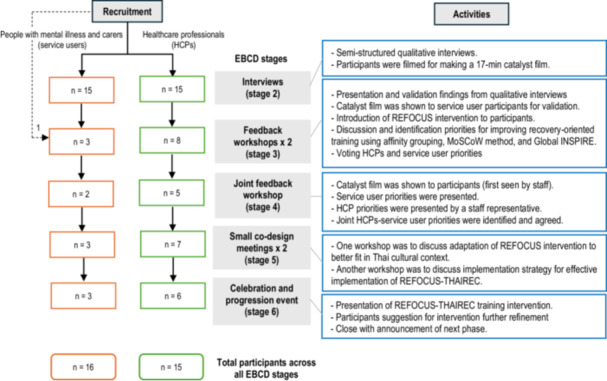
Recruitment (type and number) of participants and activities across all EBCD stages.

Figure 3 presents the recruitment process by type of participants and workshop attendance, and activities through the EBCD process.

### 3.3 Data Collection

#### 3.3.1 Gathering Experience Phase of EBCD

##### 3.3.1.1 Qualitative Interviews (Stage 2)

In the experience‐gathering phase of EBCD, participants' experiences, thoughts and perceptions of mental health recovery were collected through interviews and feedback workshops and reported elsewhere [45]. These findings informed the co‐design phase by identifying key factors influencing recovery in the Thai context (e.g., conceptions of mental health recovery, attitudes towards recovery and the factors that impede and facilitate recovery). All interviews were audio recorded, and with consent, five PwM and three carers were also filmed following the EBCD approach. The first author (N.I.) identified touchpoints from the videos, which are key moments or interactions that participants perceive as crucial to the overall experience of receiving care [43]. Using a professional video editing programme, selected interview excerpts illustrating key themes of PwM and carer experiences were compiled into a 17 min catalyst film to support discussion [48].

##### 3.3.1.2 Feedback Events (Stage 3)

Summary findings from qualitative interviews were presented in two face‐to‐face feedback workshops. Separate workshops were held for (1) PwM and their carers, who participated in the same workshop, and (2) HCPs. Participants in the PwM/carer group were shown the catalyst film to determine how well it reflected their experiences. They then discussed and identified priorities for improving recovery‐oriented practice and for adapting the REFOCUS/REFOCUS‐PULSAR training intervention using the MoSCoW (Must have, Should have, Could have, Won't have) method for prioritisation [49]. This method was selected due to its simple, intuitive structure and flexibility [50], which allows adaptation across different co‐design activities. Although its subjectivity and lack of formal criteria can lead to inconsistencies in practice, its emphasis on direct stakeholder involvement supports transparent decision‐making [51, 52].

#### 3.3.2 Co‐Design Phase of EBCD

##### 3.3.2.1 Joint Co‐Design Workshop (Stage 4)

The catalyst film was then presented to all participants at the joint workshop. The film was designed to visually present service users' experiences and help participants with similar experiences to connect, and to provide a meaningful starting point for discussion and collaborative design [53]. Four priorities of service users were presented alongside their voices from the catalyst film, and four HCPs’ priorities were presented by the staff representative. Following this, a summary of the REFOCUS/REFOCUS‐PULSAR intervention and its manual [54, 55] was introduced to all participants to familiarise them with this training intervention. Further details about the interventions are available at https://www.researchintorecovery.com/research/refocus/. Afterwards, all participants took part in facilitated discussions aimed at identifying joint priorities, again using the MoSCoW method, for improving and adapting the REFOCUS training intervention.

##### 3.3.2.2 Small Co‐Design Team Meetings (Stage 5)

Two small co‐design workshops were conducted by N.I. with the same participants. The first workshop focused on adapting the REFOCUS/REFOCUS‐PULSAR training manual to better align with the Thai cultural and service context. The second workshop explored strategies to support the effective implementation of the adapted REFOCUS‐THAIREC training. During the workshops, participants discussed potential adaptations, shared contextual insights and collaboratively generated suggestions for both content adaptation and implementation strategies. Discussions were guided by the MADI framework to support structured consideration of contextual and implementation factors [15]. Decisions about adaptations were developed collaboratively during the workshops. The lead author continued to work closely with participants throughout the co‐design process by sharing materials and draft documents reflecting the adaptations identified in each workshop for further feedback and refinement.

The ADAPT guidance suggests that involving intervention developers can help preserve an intervention's causal mechanisms during adaptation [14]. In this study, one member of the research team (M.L.) was involved in developing the original REFOCUS/REFOCUS‐PULSAR intervention and provided input during the adaptation process. Accordingly, the core components of the original training manuals were retained [56]. During the co‐design workshops, participants also discussed which elements should remain unchanged to maintain the intervention's integrity and focused on identifying contextual adaptations and additional components to support implementation in the Thai setting.

##### 3.3.2.3 Celebration and Progression Event (Stage 6)

All participants involved in stages 1 to 5 were invited to join this event. Following collaborative work with participants, the REFOCUS‐THAIREC intervention adaptations were developed and presented to the participants, who were encouraged to review and discuss both the intervention adaptation content and the proposed implementation strategy. Any suggested changes and refinements were addressed.

Finally, six members of the Patient and Public Involvement (PPI) team, including a mental health nurse, a psychiatrist, a psychologist, a peer support specialist/researcher, a person with lived experience of mental illness, and a carer, were invited to review the final REFOCUS‐THAIREC manual.

### 3.4 Data Analysis

#### 3.4.1 MoSCoW Method for Prioritisation

Priorities for improving recovery‐oriented practice and adapting the REFOCUS/REFOCUS‐PULSAR intervention were identified from both feedback events. To prioritise and quantify these priorities, we adapted the MoSCoW method [49] as a ranking approach. Participants were asked to score each priority using the MoSCoW categories: Must have = 4 (essential and critical), Should have = 3 (important but not vital), Could have = 2 (desirable but not necessary), and Won't have = 1 (not a priority at this stage). Scores were aggregated to rank eight priorities, with the four highest selected for further development in small co‐design team meetings.

#### 3.4.2 MADI Framework Analysis

The transcripts from the small co‐design workshops were uploaded to NVivo (version 15) to support data organisation, management and analysis. The first author (N.I.) conducted an independent deductive analysis guided by the MADI framework [15] and discussed with M.L. and A.G. Adaptation characteristics, including what was modified, the nature of the adaptation, and for whom the adaptation was made, were analysed in relation to the four jointly prioritised areas for intervention adaptation. In addition, mediating or moderating factors and implementation outcomes, including adoption, appropriateness, feasibility and sustainability, were examined to inform the development of implementation strategies.

## 4 Rigour and Reflexivity

To enhance rigour, this study followed established qualitative and co‐design methods with clear documentation of each EBCD stage [43, 44]. Credibility was supported through triangulation of feedback events and co‐design workshops and the involvement of diverse stakeholders [57]. Transparency was ensured through detailed reporting guided by the MADI framework [15] and regular team discussions. Reflexivity was maintained throughout. The research team had experience in mental health and the Thai context. N.I., a Thai male mental health nurse trained in EBCD, led data collection and analysis, which may have influenced interpretation. This was addressed through team reflection and efforts to minimise power imbalances using non‐hierarchical facilitation, accessible language and inclusive participation.

## 5 Results

### 5.1 Participants

A total of 31 participants contributed across the EBCD stages, with eight HCPs and four service users actively participating in the co‐design workshops that informed the intervention adaptation (see Figure 3).

### 5.2 Identified Priorities

**Figure 4:**
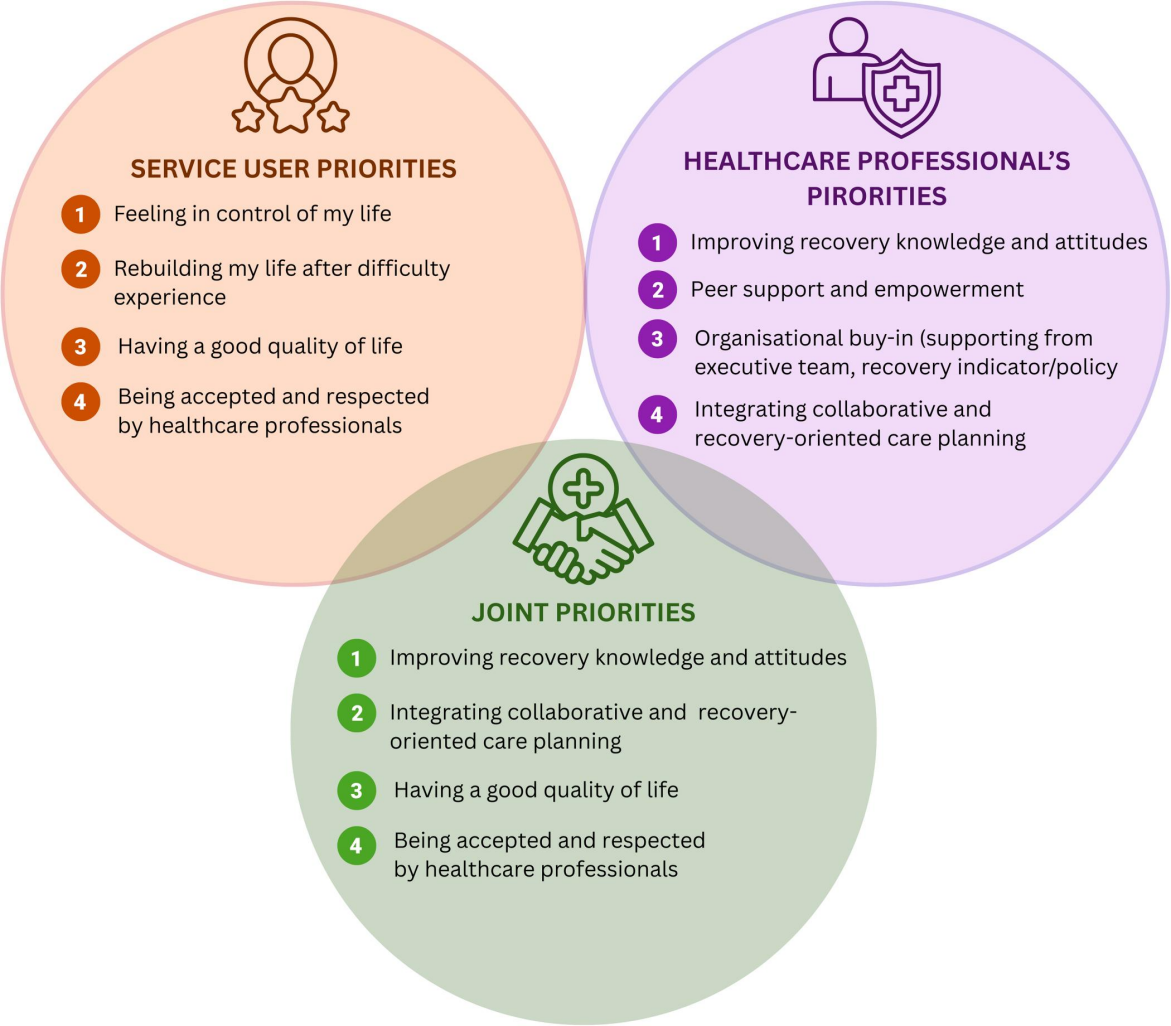
Service user, healthcare professionals and joint priorities for improving recovery‐oriented care/practice.

Figure 4 illustrates the four priorities identified separately by service users and HCPs during stage 3 of the EBCD process, as well as the shared priorities subsequently agreed during the joint workshops in stage 4.

### 5.3 REFOCUS‐THAIREC Adaptations

This section outlines the cultural adaptations of the co‐designed REFOCUS‐THAIREC training intervention. The four joint priorities for intervention adaptations identified in the joint co‐design workshop were discussed during the smaller co‐design workshops, with the use of the MADI adaptation model [15]. Supporting quotes across the adaptation and implementation strategy categories can be found in Supporting Information S4: Appendix 4.

#### 5.3.1 Priority 1: Improving Recovery Knowledge and Attitudes (Especially for Non‐Mental Health Staff)

##### 5.3.1.1 Include Non‐Mental Health Professionals and Non‐Professional Health Workers

Carers' experiential knowledge was seen as providing insights that differed from, but complemented, professional perspectives by highlighting the practical and relational aspects of recovery within everyday home life that are often less visible in clinical settings. Whereas HCPs commonly focused on symptom management and treatment adherence, carers emphasised recovery as an ongoing process shaped by family relationships, daily routines and sustained support. This difference suggested that involving people with lived experience, family members or carers as co‐trainers could strengthen the REFOCUS‐THAIREC programme by broadening HCPs' understanding of recovery beyond clinical care alone.

I think it would be possible [to include family members], and it would actually be a good thing. Because doctors [HCPs] don't really experience things directly like family members or caregivers do.

(Carer01)

A similar contrast emerged in relation to VHVs, whose role was positioned at the interface between formal services and community life. Their proximity enabled them to identify changes in wellbeing and provide continuity of support that formal services could not consistently maintain. As a result, participants supported involving peer support workers and VHVs not only as learners but also as co‐facilitators, suggesting that recovery‐oriented practice may be strengthened when knowledge is shared across professional and community boundaries.

However, participants also recognised that such involvement was shaped by local service capacity, as the availability of peer support roles and the readiness of services to involve non‐professional workers varied across settings.

##### 5.3.1.2 Simplify the Content and Familiarise Trainees With Recovery‐Oriented Practice

Conceptual unfamiliarity with recovery was identified as a key barrier to training uptake, particularly among non‐mental health workers. Participants suggested that applying recovery principles requires a basic understanding of mental health, which may be challenging for general nurses, public health officers, general practitioners, peer support workers and VHVs, whose roles are often shaped by broader public health responsibilities. As a result, participants emphasised that training content should be simplified and recovery concepts presented in clear, accessible ways to ensure relevance and engagement across both professional and non‐professional roles.

##### 5.3.1.3 Include Shared Meaning of Recovery in Thai Context

The emphasis on providing clear guidance reflected the need for a shared understanding of personal recovery among workers. Participants recognised that differing interpretations of recovery could limit consistent application in practice, particularly among staff with limited mental health training. Small group discussions were therefore viewed as a practical strategy to encourage reflection, clarify assumptions and develop a more consistent understanding of recovery‐oriented practice across participants.

##### 5.3.1.4 Illustrate With Realistic Case Studies

Participants viewed realistic case studies as important for translating recovery concepts into practical understanding. Rather than relying on abstract explanations, case examples were seen as helping trainees recognise how recovery processes align with the five domains of the CHIME framework [21] within everyday care. This reflected a need to make recovery‐oriented practice more concrete and applicable across different service settings.

At the same time, participants questioned the value of presenting only successful recovery stories, as this could create unrealistic expectations and position recovery as an ideal rather than an ongoing process. They instead preferred diverse cases that included both progress and setbacks, reinforcing the understanding of recovery as non‐linear, individual, and shaped by fluctuating experiences. This was considered more likely to support realistic expectations and meaningful engagement with recovery‐oriented practice.

##### 5.3.1.5 Incorporating a Catalyst Film

Visual narratives were identified as an effective mechanism for making lived experience visible and strengthening reflective practice among HCPs. Participants suggested that short films could translate recovery‐oriented principles into emotionally meaningful learning by exposing how routine professional behaviours may affect service users’ experiences. This was seen as particularly important for challenging taken‐for‐granted practices and fostering empathy, as visual materials created stronger emotional engagement than written examples alone.

[…] After that day, I changed the way I work. Ever since I saw that clip (catalyst film), I stopped doing it [speaking carelessly with patients].

(HCP03)

Participants also linked film‐based learning to greater awareness of contextual barriers to recovery, particularly those outside clinical settings. Showing service users within their home environments was viewed as a way to reveal practical difficulties such as travel burden, financial constraints and family responsibilities that often remain invisible in routine care. This suggested that visual narratives could support recovery‐oriented practice not only by improving interpersonal awareness, but also by helping staff situate clinical care within the broader realities of everyday life:

Many staff have never seen patients’ environments. Without that touch, treatment becomes routine. […] They don't think about how difficult it is for the patient to get there, or what the costs are.

(HCP05)

#### 5.3.2 Priority 2: Integrating Collaborative and Recovery‐Oriented Care Planning to Usual Care

##### 5.3.2.1 Provide a Recovery‐Oriented Care Plan Template

Participants positioned current care planning practices as largely incompatible with recovery‐oriented principles, describing them as primarily risk‐focused and shaped by professional judgement rather than service users’ own priorities. Care plans were commonly organised around symptom control, risk management and clinical evaluation, with limited space for service users to express personal goals or participate in decision‐making. This reflected a predominantly clinician‐led model, in contrast to recovery‐oriented care planning, which depends on shared understanding and collaborative goal setting:

In Thailand, if a patient has schizophrenia, we just list risks one to six, then evaluate. We do not see their perspective. […] But the [recovery] principle is to talk with patients and base the care plan on their problems, right?

(HCP02)

The shift towards recovery‐oriented care planning was therefore seen as requiring both greater service user involvement and stronger interdisciplinary collaboration among HCPs. Participants identified practical tools, such as structured templates and worked examples, as necessary to support this transition by making collaborative care planning more feasible in routine practice.

#### 5.3.3 Priority 3: Having a Good Quality of Life (Service Users)

##### 5.3.3.1 Provide Problem Solving and Management Skills

Participants framed good quality of life not simply as the absence of symptoms, but as the capacity to manage everyday difficulties and respond effectively to ongoing challenges. This positioned recovery as closely linked to resilience and practical functioning in daily life rather than clinical improvement alone. Participants therefore viewed coping and problem‐solving skills as important components of training, suggesting that strengthening these skills among HCPs could improve their ability to support service users in achieving more sustainable recovery and better overall quality of life.

##### 5.3.3.2 Create or Facilitate a Supportive Environment

Participants positioned meaningful activities and broader life experiences as central to recovery, arguing that wellbeing could not be sustained through symptom management alone. Recovery was understood as being shaped by everyday living conditions, routines, and opportunities for engagement, with activities such as watering plants, growing vegetables, travelling, or experiencing new environments seen as important sources of purpose and emotional stability. These experiences were described as offering alternative ways of experiencing happiness, particularly for individuals whose sense of wellbeing had previously been closely associated with substance use.

#### 5.3.4 Priority 4: Being Accepted and Respected by HCPs

##### 5.3.4.1 Provide Recovery Language Guide

Participants identified language as a key mechanism through which recovery‐oriented care is either supported or undermined. Communication was not viewed as neutral, but as shaping service users’ identities, influencing therapeutic relationships, and reinforcing either dignity or stigma. Terms perceived as blaming or labelling, such as ‘crazy’, (HCP01), were seen as contributing to shame and social exclusion, suggesting that language itself can become a barrier to recovery.

In contrast, positive and respectful communication, including open‐ended questions, encouragement and praise, was understood as strengthening trust and engagement in care. Participants also raised cultural concerns around diagnostic labels such as schizophrenia, questioning whether such terms may carry particularly harsh meanings within the Thai context. This extended to families, where insensitive communication from staff could negatively affect both carers and service users.

### 5.4 Implementation Strategy for REFOCUS‐THAIREC

This section outlines the potential implementation strategies of the REFOCUS‐THAIREC training intervention guided by the MADI model [15] (see Table 1).

**Table 1:** The co‐design discussion on REFOCUS‐THAIREC adaptations and implementation strategies.

Priorities for adaptations	Possible action	Proposed adaptation attributes/ Implementation strategies
*Priorities for adaptations*		
Priority 1: Improving recovery knowledge and attitudes (especially for non‐mental health workers)	• Simplify the content of recovery‐related knowledge for non‐mental health professionals (general nurse, public health officer, general practitioner, etc.) and non‐professional health workers (peer support worker, VHVs) to reflect the integration of mental health into primary and public health services in Thailand	‐Avoid using jargon and simplify word
		‐Provide a clear meaning and shared meaning of ‘personal recovery’
		‐Provide at least two contrasting case study examples of people's recovery journey
		‐An interactive open online version of the training course could help refresh knowledge as staff in sub‐district hospital often do not work with people with mental health conditions
	• Improve recovery attitudes of health care providers	‐Provide a film containing emotional touchpoints of service user's experience and thoughts
	• Equip non‐mental health staff with some basic skills to improve confidence in providing effective recovery‐oriented care	‐Providing pro‐recovery skills for communicating with people living with mental health conditions
		‐Provide a checklist of the core elements needed for providing recovery‐oriented care
Priority 2: Integrating collaborative and recovery‐oriented care planning within usual care	• Make the care plan to be more person‐centred, with shared decision‐making and shared responsibility (as Thai care planning is typically clinician‐led)	‐Provide a recovery‐oriented care plan template ‐Provide an example of recovery‐oriented care plan formulation for training purposes
	• Ensure discussion and agreement of HCPs’ expectations of service user recovery to ensure realistic goals, and to prevent over‐expectation and undue pressure on service users (as Thai care plan usually be clinician led)	
Priority 3: Having a good quality of life	• Ensure that healthcare workers have positive attitudes towards improving service user's recovery and quality of life	‐Encourage healthcare workers to better understand service users and facilitate discussions with them about their strengths ‐Promote the self‐worth and pride of service users
	• Promote a supportive environment	‐Assess service users’ houses and wider environments through home visits and making therapeutic suggestions ‐Encourage service users to try going to new places to find new inspiration
	• Support service users to be able to solve problems in daily life which could help reduce stress and anxiety and bring about a good quality of life	‐Provide/discuss problem‐solving skills and management strategies with service users
	• Suggest healthcare workers provide support to service users to manage financial and transport issues	‐Provide information/suggestions on managing their daily lives, e.g., saving strategies, coordinating with social care, coordinating with occupational centres
Priority 4: Being accepted and respected by healthcare workers	• Move towards trauma‐informed care.	‐Introduce the basic concept of recovery language ‐Provide recovery language guide to healthcare workers
	• Ensure service users’ voices are being heard.	
	• Promote the use of recovery language (as the Thai language is usually sensitive in terms of mental health aspects)	
*Potential mediators*		
**Alignment with core functions/relationship to fidelity:** Adaptation consistent with core functions of the intervention or implementation strategy?	• Providing a refreshing course to boost recovery knowledge and attitudes, e.g., a short open online course that is easily accessible	‐Increasing accessibility by making it easier to access and less complex
	• Include managers and executive team members in the Thai REFOCUS developing/revising process • Invite managers and executive team members to participate in Thai REFOCUS training	‐Support from manager or executive team
	• Provide support and minimise paperwork for recovery‐oriented care	‐Ensure that recovery‐oriented care does not put burden on non‐mental health staff who are also responsible for physical health and infection control
*Potential moderators*		
**Goal/Reason for adaptation:** Adaptation made for a reason/goal that addresses fit? **Systematic:** Adaptation made with due consideration given to impact on outcomes and using a systematic process (consulting data, stakeholders, theory, best practice)? **Proactive:** Adaptation made due to anticipated obstacle	• Implement recovery outcome measure in practice *(e.g., applying Thai Global INSPIRE during care planning process and/or using QPR measure to assess recovery outcomes)*	‐Improve clarity on the outcomes of recovery‐oriented care ‐Ensure a valid recovery outcome measure is in place and used as an indicator of the success of recovery‐oriented care
*Proposed implementation strategies*		
Adoption (Uptake; utilisation; initial implementation; intention to try)	• Providing a short video advertisement to introduce REFOCUS‐THAIREC in an interesting way.	‐Consider that staff might lack understanding and feel worried/unsure about recovery‐oriented care/practice, which is quite new in Thailand. ‐Make them aware of the importance of training and recovery‐oriented care to make it easier for them to engage in training
	• Offer additional mental health skills training alongside REFOCUS‐THAIREC (for non‐mental health workers)	
Appropriateness (perceived fit; relevance; compatibility; suitability; usefulness; practicability)	• Trainers: should include professionals, peer support workers, VHVs, staff who have lived experience with a mental health condition, and carers • Offer peer support workers, VHVs, and carers additional skills before joining as a trainer, e.g., communication skills	‐Lived experience of mental health condition or recovery journey should be brought to the training through appropriate selection of trainers. ‐Lived experience trainers such as peer support workers, VHVs, carers should be well prepared before support delivering the training
	• Trainees: should include mental health and non‐mental health staff, and non‐professionals such as peer support workers and VHVs • Offer separate sessions for HCPs and non‐professional trainees due to the diversity of their educational background and experience	‐Trainees should include nonprofessional staff (peer support workers and VHVs) as they are directly and closely involved with service user care within community. ‐Consider the needs of different types of trainees when organising and scheduling training
Feasibility (actual fit or utility; suitability for everyday use; practicability)	• Have gatekeepers who work in related institutions to help coordinate with stakeholders and funders • Secure funding to run the training (from the Ministry of Public Health, local authority etc.)	‐ Secure funding by highlighting the importance and impact of recovery training to service users
	• Test effectiveness of the training through randomised control trials to secure further funding	
Sustainability (maintenance; continuation; durability; incorporation; integration; institutionalisation; sustained use; routinisation;)	• Make training publicly available	‐Provide accessible refresher training (e.g., online)
	• Offer a certificate after training	
	• Provide online modules and refreshing training. • Provide additional recovery care/practice consultation after training	
	• Ensure that recovery outcome measures are implemented into routine practice	
	• Select and support a recovery ‘champion’ within the team	

Abbreviations: HCP, healthcare professional; VHV, village health volunteer.

#### 5.4.1 Potential Mediators

##### 5.4.1.1 Accessibility and Simplicity of Training

Participants identified implementation success as dependent on three interrelated conditions: usability, comprehensibility and accessibility. Training and associated tools needed to be sufficiently user‐friendly to accommodate differences in age, educational background and professional role, particularly within a workforce that included both HCPs and non‐professional workers. Content clarity was considered especially important for older trainees, such as carers and VHVs, whose engagement may be limited if training is overly complex or academically framed. In addition, ease of access was viewed as necessary for promoting uptake and supporting routine use in practice.

##### 5.4.1.2 Support From Manager and Executive Team

Participants identified managerial understanding and executive support as central to implementation success, suggesting that recovery‐oriented training could not be sustained through staff motivation alone. Organisational leadership was seen as shaping whether new initiatives were prioritised, resourced and embedded into routine practice. Participant's previous experiences of unsuccessful programmes illustrated that even well‐designed interventions could fail when managers did not recognise their purpose or provide institutional support, particularly in community settings. In contrast, when managers understood the value of recovery‐oriented practice, they were perceived as more likely to facilitate staff participation, allocate necessary resources and support longer‐term sustainability.

##### 5.4.1.3 Limiting Extra Burden From Recovery‐Oriented Care

Participants positioned paper‐based care planning as a practical limitation to implementing recovery‐oriented practice, particularly in community settings where continuity of care depends on mobility and timely information sharing. Paper documentation was seen as restricting communication across staff and increasing administrative burden, which could undermine collaborative planning. Digital alternatives, such as smartphone‐accessible online platforms, were therefore viewed as a way to improve efficiency, support clearer reporting and strengthen continuity during home visits and follow‐up care.

At the same time, participants recognised that digital approaches required careful consideration of local governance and confidentiality requirements. These tools were not intended to replace formal clinical records, but to function as practical supports for collaborative care planning.

#### 5.4.2 Potential Moderators

##### 5.4.2.1 The Implementation of Recovery Outcome Measure in Practice

Participants identified the presence of formal outcome measures as an enabler of implementing recovery‐oriented practice, as progress was often difficult to define using existing clinical indicators alone. Without clear measures, recovery‐oriented care risked remaining conceptually valued but difficult to evaluate in practice. Assessment tools were therefore seen as necessary to make recovery outcomes more visible and to provide evidence of whether recovery‐oriented approaches were being applied effectively.

#### 5.4.3 Adoption

##### 5.4.3.1 Provide Additional Mental Health Skills (for Non‐Mental Health Workers)

Participants identified limited exposure to mental health training as a factor reducing confidence in delivering recovery‐oriented care, particularly in complex situations such as responding to suicidal ideation. This suggested that competence in recovery‐oriented practice depended not only on individual knowledge, but also on organisational support through supervision, guidance and opportunities for applied learning. Managerial support was therefore viewed as necessary for strengthening both staff confidence and the consistent delivery of recovery‐oriented care.

Training methods based on real‐life examples and case‐based scenarios were considered more effective than lecture‐based teaching for developing practical skills. Participants emphasised the need for concrete guidance on responding to suicidal thoughts, conducting risk assessment and providing ongoing support, as these situations often created uncertainty in routine practice. Interactive and experiential approaches, including real stories, video scenarios and reflective discussion, were therefore seen as more likely to support the translation of recovery principles into everyday clinical communication and decision‐making.

#### 5.4.4 Appropriateness

##### 5.4.4.1 Preparing Lived Experience Trainers

Participants identified lived experience as a valuable form of knowledge within recovery‐oriented training, as it provided perspectives that could not be fully captured through professional teaching alone. Involving peer support workers, staff with lived experience, VHVs, and carers was seen as a way to make recovery principles more credible and practically relevant by linking abstract concepts to real experiences of living with and supporting mental health recovery.

At the same time, participants recognised that lived experience contributions required careful preparation. Sharing personal stories without sufficient understanding of recovery principles risked reducing their educational value, whilst the emotional sensitivity of discussing past distress raised concerns about potential harm. This suggested that the inclusion of lived experience depends on structured preparation and clear guidance, ensuring that contributions are both meaningful for learning and safe for those involved.

##### 5.4.4.2 Offer Separate Sessions for HCPs vs. Non‐Professional Trainees

Differences in professional roles, educational backgrounds and organisational hierarchy shaped how participants viewed the structure of training delivery. Separate sessions for professional and nonprofessional groups were considered more appropriate, as participants believed that a single training format would not adequately address differences in knowledge, confidence and prior experience with mental health concepts. This was particularly evident for VHVs, whose diverse ages, educational levels and limited exposure to formal mental health training influenced both learning needs and participation styles.

Hierarchical relationships were also seen as affecting engagement during training. Mixed professional groups were perceived as potentially limiting open discussion, as nonprofessional participants and junior staff might feel reluctant to speak freely in the presence of senior professionals. This suggested that separating groups was not only a practical adaptation to different learning needs, but also a way to create safer spaces for participation and reflection.

#### 5.4.5 Feasibility

##### 5.4.5.1 Secure Funding

The implementation of recovery‐oriented training was framed as contingent on broader structural support rather than the training programme itself. Participants positioned endorsement from the Ministry of Public Health as a key condition for legitimacy, sustainability and integration into routine practice, reflecting the influence of top‐down governance within the Thai health system. In this context, local pilot programmes were viewed as a necessary first step, with evidence of effectiveness required before wider institutional backing and funding could be secured.

#### 5.4.6 Sustainability

##### 5.4.6.1 Provide Online Modules and Refresher Training

Sustaining recovery‐oriented practice was understood as requiring continuous learning rather than one‐off training events. Participants viewed flexible and ongoing learning opportunities, particularly through online modules, as important for reinforcing knowledge and supporting refresher learning over time. Compared with traditional face‐to‐face training, which was considered costly and resource‐intensive, online formats were seen as more practical and accessible, allowing staff to revisit content at their own pace and integrate learning into routine professional development.

## 6 Discussion

This study provides insight into the cultural adaptation and implementation of recovery‐oriented training (REFOCUS/REFOCUS‐PULSAR) in Thai primary and community mental healthcare services. Four key content adaptations were recommended: a catalyst film, a recovery‐oriented care planning component, two case studies illustrating recovery journeys and a recovery‐oriented language guide. Six implementation strategies were identified: experiential and contextually relevant learning, digital delivery options, preparation for lived experience trainers, separate training streams for professional and non‐professional groups, practical recovery care planning tools and the use of recovery outcome measures. The findings suggest that implementing recovery‐oriented practice in Thailand requires more than adapting training content. It also involves addressing broader organisational, relational and professional norms within a system shaped by biomedical models, professional hierarchy, family involvement and community‐based care. This study contributes to international implementation literature by demonstrating how recovery‐oriented interventions developed in Western settings require substantial contextual adaptation in hierarchical and non‐Western mental health systems.

A central insight from this study is the importance of experiential and interactive learning. Participants emphasised that abstract concepts such as personal recovery remain relatively unfamiliar in Thai mental healthcare and are best conveyed through practical examples, case‐based scenarios, visual materials and a clearly structured recovery care plan. Importantly, the findings suggest that implementing recovery‐oriented practice in Thailand may represent more than the introduction of a new training intervention. Rather, recovery‐oriented approaches challenge historically dominant biomedical, clinician‐led and risk‐focused models of mental healthcare by repositioning service users as active partners within care planning and decision‐making processes. In this sense, the implementation of REFOCUS‐THAIREC may require broader cultural and organisational shifts in professional identity, power relations and understandings of expertise within Thai mental health services. This aligns with international evidence that participatory, contextually relevant learning is more effective than purely didactic instruction [58, 59].

Tailoring training to participants’ backgrounds was another important theme. Separate sessions for professional and non‐professional staff, including VHVs, were suggested to accommodate differences in education, experience and confidence, and to encourage open communication [60]. The inclusion of VHVs and non‐professional workers also reflects broader international shifts towards task‐sharing approaches within low‐resource mental healthcare systems, whereby responsibilities traditionally associated with specialist mental health professionals are redistributed across community‐based and lay workforces to improve accessibility, continuity of care, and workforce capacity [61, 62]. Within the Thai context, involving VHVs as both trainees and potential co‐facilitators highlights how recovery‐oriented practice may depend upon collaborative workforce models that extend beyond specialist mental health services. The findings also suggest that hierarchical organisational cultures may inhibit the implementation of collaborative recovery‐oriented practice. Participants described how mixed‐group training environments could unintentionally reproduce professional hierarchies, silence and deference, particularly where non‐professional workers or junior staff felt reluctant to speak openly in the presence of senior clinicians. This suggests that implementation strategies themselves required cultural adaptation, with separate training streams functioning not only as educational adjustments, but also as mechanisms to support psychologically safer and more participatory learning environments.

The integration of lived experience into recovery training emerged as a pivotal element. Involving peer support workers, carers and staff with personal mental health experience was thought to enhance the authenticity and relevance of training. However, preparation is critical, and participants agreed that recovery‐focused education should precede storytelling to ensure safe, accurate and meaningful contributions. These findings mirror previous studies highlighting the benefits of lived experience in recovery education whilst noting the need for structured support [63, 64]. Providing practical tools, such as recovery care planning templates, recovery outcome measures (e.g., Global INSPIRE [65], QPR [66]), a recovery language guide, and exposure to service users’ real‐life contexts, were also seen as essential to bridge theory and practice for HCPs and VHVs. These approaches may support staff in internalising recovery principles and applying them confidently, reflecting the dynamic and individualised nature of recovery [67].

Finally, systemic and organisational support was identified as crucial. Managerial endorsement, institutional backing and sustainable funding were viewed as prerequisites for successful implementation. This aligns with a previous study showing that strategic alignment across technical, political and institutional domains can enable transformational policy change [68]. However, in real‐world low‐recourse settings, Thailand faces staff shortages and heavy workloads, particularly in mental health services [11], which may limit HCPs’ capacity to engage in or implement recovery‐oriented practice. In addition, mental health care in Thailand often prioritises early detection and crisis intervention [69], with limited emphasis on recovery and rehabilitation, potentially constraining attention and resources for recovery‐focused initiatives. Pilot programmes and rigorous evaluation, including the use of recovery outcome measures, may help generate the evidence required to secure policy support. Flexible strategies, such as online modules, refresher sessions, and regular reflective practice, may further reinforce skills and promote continuous professional development.

### 6.1 Strengths and Limitations of the Work

This study has several important strengths. By using a participatory co‐design approach, it actively involved Thai service users and HCPs in shaping the recovery‐oriented training intervention, ensuring that the adaptation process was contextually relevant, practical and grounded in lived experience. Including participants from a range of community and primary care settings enabled a richer understanding of how recovery‐oriented practice may be implemented across diverse service contexts. The integration of the ADAPT guidance, MADI model and CHIME recovery framework added conceptual and methodological rigour to both the adaptation and implementation planning processes.

However, several limitations should be acknowledged. First, as this study focused on cultural adaptation and co‐design, it did not evaluate the long‐term implementation, fidelity, sustainability or clinical and recovery outcomes of REFOCUS‐THAIREC. Consequently, further research is required to examine the intervention's effectiveness and implementation in real‐world settings before wider scale‐up can be recommended. Second, the findings are specific to rural district and subdistrict healthcare settings in Chiang Mai, and both the cultural context (including Buddhist values, VHV infrastructure and NANDA‐based care planning) and organisational structures may differ across other Thai regions and international settings. As such, further contextual and cultural adaptation may be required before transferability to other healthcare systems can be assumed. Power imbalances between professional and non‐professional participants within co‐design workshops also represent an important limitation. Although efforts were made to support inclusive participation through non‐hierarchical facilitation techniques, accessible language and collaborative discussion methods, hierarchical professional structures prevalent within Thai healthcare may nevertheless have constrained the contributions of service users and carers during joint and small co‐design workshops involving HCPs. Notably, the subsequent recommendation for separate training sessions for professional and non‐professional groups partly emerged in response to these dynamics. The focus on tailoring the intervention to the needs of Thai healthcare workers may also limit the generalisability of some findings to implementation work within other healthcare contexts, and the intervention would require further cultural or systemic adaptation before being applied in other countries. Finally, although participant numbers were relatively small, this is consistent with co‐design methodologies, where smaller groups are typically used to facilitate deeper engagement, iterative discussion and collaborative intervention development [70, 71].

### 6.2 Implications for Research, Policy and Practice

Further research is needed to evaluate the REFOCUS‐THAIREC in terms of implementation and long‐term outcomes in real‐world settings. Collaborative studies involving policymakers, practitioners and people with lived experience could continue to inform the refinement of this and similar recovery interventions, and future work could support intervention adaptation for use in a range of contexts including pre‐registration training, prisons, shelters and inpatient services. A participatory approach would ensure that future research remains grounded in local realities and consistently aligned with the values of recovery‐oriented care.

Our findings recognise a strong need for policy‐level endorsement to ensure the sustainability and scalability of recovery‐oriented initiatives. Integrating recovery principles within existing national mental health strategies and professional development frameworks could promote a unified direction across services. Policymakers should also consider embedding the training within provincial or national mental health programmes, allocating dedicated funding and identifying local champions to coordinate and maintain momentum. Aligning the intervention with broader policy priorities such as national mental health policy and strategy, well‐being and public health promotion and ageing populations could strengthen its relevance and feasibility for national adoption.

At the practice level, we underscore the need for continued training and reflective opportunities for HCPs to internalise and apply recovery principles in their daily work. Establishing recovery champions within teams could facilitate ongoing peer learning and sustain practice change. Integrating training materials into electronic or online platforms would enhance accessibility, promote self‐directed learning and reduce reliance on in‐person delivery.

## 7 Conclusion

This study offers guidance for adapting and implementing co‐designed recovery‐oriented training (REFOCUS‐THAIREC) for healthcare workers in Thai community settings. By involving both service users and HCPs, it identifies contextually relevant strategies, including experiential learning, structured incorporation of lived experience and tailored approaches for professionals and non‐professional groups. Organisational support, policy endorsement and flexible delivery methods, such as online refresher modules, are highlighted as essential enablers. Although the intervention has not yet been implemented or evaluated, these findings provide a practical foundation for future piloting, integration into national and provincial mental health programmes, and the advancement of person‐centred, recovery‐oriented care. More broadly, this study demonstrates how recovery‐oriented interventions developed in Western contexts can be systematically co‐designed and culturally adapted for non‐Western mental health systems whilst retaining core recovery principles and responding meaningfully to local cultural, organisational and workforce realities.

## Author Contributions

**Natthapon Inta:** conceptualisation, writing – original draft, methodology, formal analysis, data curation, project administration, writing – review and editing, visualisation, investigation. **Annmarie Grealish:** conceptualisation, methodology, supervision, writing – review and editing, validation. **Mary Leamy:** conceptualisation, methodology, validation, supervision, writing – review and editing.

## Ethics Statement

Ethical approval for this study was obtained from King's College London (Reference number HR/DP‐24/25‐45539) and Chiang Mai Provincial Public Health Office, Thailand (CM0033.010/2721), and all participants provided written informed consent.

## Conflicts of Interest

The authors declare no conflicts of interest.

## Supporting information

Supporting File 1

Supporting File 2

Supporting File 3

Supporting File 4

## Data Availability

The data that support the findings of this study are available on request from the corresponding author. The data are not publicly available due to privacy or ethical restrictions.
