# Supplementary material for: Cultural Adaptation and Implementation Strategy of a Recovery‐Oriented Mental Health Training Intervention (REFOCUS‐THAIREC) for Healthcare Workers in Thailand: An Experience‐Based Co‐Design
Source: Health Expect. 2026 Jun 22;29(3):e70738. doi: 10.1111/hex.70738 (PMC13287322; doi:10.1111/hex.70738)
Supplement: Supplementary file 1 — Supporting File 1 [file HEX-29-e70738-s002.docx]

**Appendix 1: Co-design workshop discussion guides**

**The co-design discussion on REFOCUS-THAIREC adaptations, guided by MADI**

| **The adaptation characteristics according to priorities** | **Prompt questions with lay language used with co-design participants** |
| --- | --- |
| **Priority 1: Improving recovery knowledge and attitudes (especially for non-mental health staff)** | |
| **What is modified**  (content, delivery, training and evaluation, implementation and scale-up activities) | **Content (What is taught or included)**   - How to simplify recovery knowledge session for non-mental health staff and non-HCP (peer supporter, VHVs) to understand? (e.g., CHIME framework guidance) - How do you think examples or case stories about recovery matched Thai culture or real situations in Thai services would help improve understanding? (e.g., film presentation, recovery skill set)   **Delivery (How it is taught or shared)**   - What activities, group discussions, or materials would help improve trainee’s understanding? (Apart from the main training: reflective feedback, supervision etc.) - Any open online course (MOOC) / Webinar / seminar / workshop - How to integrate lived experience to the training (HCP with mental condition/use services?) |
| **Nature of adaptation** (adding/skipping/substituting elements, shortening/condensing pacing, repeating element) | **Adding:**   - Should any new topics or examples or case study about recovery be added to help non-mental health staff understand better?   - If yes, what should be added and how? - Should extra activities or discussions be included to make the ideas of recovery clearer?   - If yes, what should be added/included and how? |
| **For whom/what is the adaptation made**  (individual, target intervention group, cohort/individuals that share a particular characteristic, individual practitioner, clinic/unit, organisation, network/system community) | - How do you think would help simplify recovery knowledge for easier understanding of non-mental health professionals and non-professionals (peer supporter, VHVs) |
| **Priority 2: Integrating collaborative and recovery-oriented care planning to usual care** | |
| **Nature of adaptation** (adding/skipping/substituting elements, shortening/condensing pacing, repeating element) | - Do you think what is the principle/elements of recovery care plan? (shared decision making/responsibility, person-centred) - What would help the increased use of shared decision-making approach? - How do you want recovery-oriented care plan to look like? - How do you want recovery-oriented care plan to be structured? - What do you want to see in recovery-oriented care plan? - What components you would like to see in the recovery-oriented care plan? - How do you want recovery-oriented care plan session to be taught? - How do you want recovery-oriented care plan to be delivered in real practice? - Any record form to help formulated care plan? |
| **For whom/what is the adaptation made**  (individual, target intervention group, cohort/individuals that share a particular characteristic, individual practitioner, clinic/unit, organisation, network/system community) | - Who do you want the recovery-care plan to be designed for use? (any specific role/professions, any specific organisation/unit/department) - Who do you want to be involved in recovery-oriented care planning? (multidisciplinary team: health care sector, social care sector, local administration, police, villagers etc.) |
| **Priority 3: Having a good quality of life** | |
| **Nature of adaptation** (adding/skipping/substituting elements, shortening/condensing pacing, repeating element) | - What things make your life feel good or satisfying right now? - What steps could help you get closer to that life? - What would help you to have a good quality of life? - What kind of support would help you maintain a good quality of life? - What changes to the training would help staff and service users understand and improve quality of life better? - How do you think the training would help you to have a good quality of life? (specifically for service users) |
| **Priority 4: Being accepted and respected by healthcare professionals** | |
| **Nature of adaptation** (adding/skipping/substituting elements, shortening/condensing pacing, repeating element) | - What makes service users feel being respected? - What make staff more listen to service users’ voices? - What make forming trust between staff and service users? (more open minded) - How staff-patients interactions look like to support recovery? (coaching, recovery language, share decision-making, style of working). |

**The co-design discussion on REFOCUS-THAIREC implementation strategies, guided by MADI**

| **The possible mediator/moderator and proposed implementation strategies** | **Prompt questions with lay language used with co-design participants** |
| --- | --- |
| **Potential mediator** | |
| **Alignment with core functions/ relationship to fidelity:** Adaptation consistent with core functions of the intervention or implementation strategy? | - Which parts of the training have the biggest effect on staff or service users? - How do supportive leaders or managers help the training work better? - How can we make sure the main ideas and skills from the training are used in daily work? |
| **Potential moderators** | |
| **Goal/Reason for adaptation:** Adaptation made for a reason/goal that addresses fit? | - How do staff experience or background make a difference in how well the training works? - Does the amount of support from managers affect how well the training works? - Are there community or cultural factors that influence how well it works? - Can you think of situations where the training would work really well? What was special about them? - Can you think of situations where the training would not work so well? What might have caused that? |
| **Systematic:** Adaptation made with due consideration given to impact on outcomes **and** using a systematic process (consulting data, stakeholders, theory, best practice)? |  |
| **Proactive:** Adaptation made due to anticipated obstacle |  |
| **Proposed implementation strategies** | |
| **Adoption**  (Uptake; utilisation; initial implementation; intention to try) | - How do you feel about this training will be using in your work? - What makes you interested (or not interested) in using REFOCUS-THAIREC? - What might make it easier/harder for people to join the training? |
| **Appropriateness**  (Perceived fit; relevance; compatibility; suitability; usefulness; practicability) | - Do you think this training fits the needs of your service? Why or why not? - What changes would make this training fit better with your needs? - Do you think the timing, style, or format is right for your team? (if not want to you want it to look like?) - Do you think when is the best time to conduct the training? |
| **Feasibility**  (Actual fit or utility; suitability for everyday use; practicability) | - Do we have the staff, time, and resources to make it happen? - Would we need extra training or support to make it work? - Who would be able to lead or run the training here? (who should be the trainer?) - What might make it difficult to use this training in your service? - What might help us to make it work more easily? - Are there rules, policies, or systems that might make it hard to do? |
| **Sustainability**  (Maintenance; continuation; durability; incorporation; integration; institutionalisation; sustained use; routinisation;) | - Can we keep using this training over the long term? Why or why not? - What might make it hard to keep the training going? - Will we have enough staff, time, and money to keep it running? - Do we want to have ongoing support or follow-up after the initial training? - Are there policies or systems that would help it stay in place? - What things would make it easier to keep the training going? |

**Note:** The questions were informed by findings from the previous experience-gathering phase (qualitative interviews and feedback workshops) of this EBCD studies and guided by MADI framework for adaptations and implementation outcomes.
