# Supplementary material for: Cultural Adaptation and Implementation Strategy of a Recovery‐Oriented Mental Health Training Intervention (REFOCUS‐THAIREC) for Healthcare Workers in Thailand: An Experience‐Based Co‐Design
Source: Health Expect. 2026 Jun 22;29(3):e70738. doi: 10.1111/hex.70738 (PMC13287322; doi:10.1111/hex.70738)
Supplement: Supplementary file 2 — Supporting File 2 [file HEX-29-e70738-s004.docx]

**Appendix 2: The co-design discussion on REFOCUS-THAIREC adaptation guided by MADI model**

| **The MADI adaptation characteristics  according to priorities** | **Possible action** | **Proposed REFOCUS-THAIREC adaptation attributes  by co-design participants** |
| --- | --- | --- |
| Priority 1: Improving recovery knowledge and attitudes (especially for non-mental health workers)   - What is modified - Nature of adaptation - For whom what is the adaptation made | - Simplify the content of recovery-related knowledge for non-mental health professionals (general nurse, public health officer, general practitioner etc.) and non-professionals health workers (peer support worker, village health volunteer) | - Avoid using jargon and simplify word use to make it suitable for non-professional trainees (peer support workers, village health volunteers).  - Provide a clear meaning and shared meaning of ‘personal recovery’. A small group discussion on the meaning of ‘personal recovery’ during the training could be considered to achieve this.  - Provide at least two contrasting case study examples of people’s recovery journey. For example:  (1) a recovery journey with good recovery outcomes according to CHIME framework.  (2) a journey which might not be perfect but still shows that a person could recover and live their life. This might help trainee to be realistic but still optimistic, ensuring they do not lose motivation.  - An interactive open online version of the training course could help refreshing knowledge as staff in sub-district hospital often do not work with people with mental health conditions. Could be considered as a supplementary course to core training. |
|  | - Improve recovery attitudes of health care providers | - Provide a film containing emotional touchpoints of service user’s experience and thoughts. The film should:  - provide the reality context of their living houses, community, and treatment journey.  - have some material that reflects recovery support service user received from staff.  - have some material reflecting service users’ expectations for their recovery and recovery support. |
|  | - Equip non-mental health staff with some basic skills to improve confidence in providing effective recovery-oriented care | - Providing pro-recovery skills for communicating with people living with mental health conditions. This could be an example/video to demonstrate examples of pro-recovery communication between staff and service users. Could be considered as a supplementary course.  - Provide a checklist of the core elements needed for providing recovery-oriented care. |
| Priority 2: Integrating collaborative and recovery-oriented care planning within usual care   - What is modified - Nature of adaptation - For whom/what is the adaptation made | - Make the care plan to be more person-centred, with shared decision-making and shared responsibility - Ensure discussion and agreement of healthcare professionals’ expectations of service user recovery to ensure realistic goals, and to prevent over-expectation and undue pressure on service users. | - Provide a recovery-oriented care plan template  - Recovery-oriented care plan potential components should include:  - Service users expectation and goals  - Clear treatment process/plan  - Service user strengths  - Current struggles  - Protective factors  - Additional service user support needs (e.g., financial, transport)  - Contact number of service user supporters  - Provide an example of recovery-oriented care plan formulation for training purposes. |
| Priority 3: Having a good quality of life   - Nature of adaptation | - Ensure that healthcare workers have positive attitudes towards improving service user’s recovery and quality of life | - Encourage healthcare workers to better understand service users and facilitate discussions with them about their strengths  - Promote the self-worth and pride of service users |
|  | - Promote a supportive environment | - Assess service users’ houses and wider environments through home visits and making therapeutic suggestions  - Encourage service users to try going to new places to find new inspiration |
|  | - Support service users to be able to solve problems in daily life which could help reduce stress and anxiety bring about a good quality of life | - Provide/discuss problem solving skills and management strategies with services users |
|  | - Suggest healthcare workers provide support to service users to manage financial & transport issues | - Provide information/suggestions on managing their daily lives e.g., saving strategies, coordinating with social care, coordinating with occupational centres |
| Priority 4: Being accepted and respected by healthcare workers   - Nature of adaptation | - Move towards trauma-informed care - Ensure service users’ voices are being heard - Promote the use of recovery language | - Introduce the basic concept of recovery language, which includes:  - The use of open questions  - Giving compliments  - Using positive or non-stigmatising words  - Being aware of non-verbal language e.g., facial expression, tone of voice, gestures  - Provide recovery language guide to healthcare workers |

**Note:**

- **What is modified** (content, delivery, training and evaluation, implementation and scale-up activities).
- **Nature of adaptation** (adding/skipping/substituting elements, shortening/condensing pacing, repeating element).
- **For whom / what is the adaptation made** (individual, target intervention group, cohort/individuals that share a particular characteristic, individual practitioner, clinic/unit, organisation, network/system community).
