# Supplementary material for: Cultural Adaptation and Implementation Strategy of a Recovery‐Oriented Mental Health Training Intervention (REFOCUS‐THAIREC) for Healthcare Workers in Thailand: An Experience‐Based Co‐Design
Source: Health Expect. 2026 Jun 22;29(3):e70738. doi: 10.1111/hex.70738 (PMC13287322; doi:10.1111/hex.70738)
Supplement: Supplementary file 3 — Supporting File 3 [file HEX-29-e70738-s001.docx]

**Appendix 3: The co-design discussion on REFOCUS-THAIREC implementation strategy guided by MADI model**

| **The MADI implementation strategies** | **Proposed REFOCUS-THAIREC implementation strategies attributes by co-design participants** | **Possible action** |
| --- | --- | --- |
| **Potential mediators** | | |
| **Alignment with core functions/ relationship to fidelity:** Adaptation consistent with core functions of the intervention or implementation strategy? | - Increasing accessibility by making it easier to access and less complex | - Providing a refreshing course to boost recovery knowledge and attitudes e.g., a short open online course that is easily accessible |
|  | - Support from manager or executive team | - Include managers and executive team members in the Thai REFOCUS developing/revising process - Invite managers and executive team members to participate in Thai REFOCUS training |
|  | - Ensure that recovery-oriented care does not put burden on non-mental health staff who are also responsible for physical health and infection control | - Provide support and minimise paperwork for recovery-oriented care |
| **Potential moderators** | | |
| **Goal/Reason for adaptation:** Adaptation made for a reason/goal that addresses fit?  **Systematic:** Adaptation made with due consideration given to impact on outcomes **and** using a systematic process (consulting data, stakeholders, theory, best practice)?  **Proactive:** Adaptation made due to anticipated obstacle | - Improve clarity on the outcomes of recovery-oriented care. - Ensure a valid recovery outcome measure is in place and used as indicator of the success of recovery-oriented care | - Implement recovery outcome measure in practice *(e.g., applying Thai Global INSPIRE during care planning process and/or using QPR measure to assess recovery outcomes)* |
| **Proposed implementation strategies according to MADI model** | | |
| **Adoption**  (Uptake; utilisation; initial implementation; intention to try) | - Consider that staff might lack of understanding and feel worried/unsure about recovery-oriented care/practice which is quite new in Thailand. - Make them aware of the importance of training and recovery-oriented care to make it easier for them to engage in training | - Providing a short video advertisement to introduce REFOCUS-THAIREC in an interesting way - Offer additional mental health skills training alongside REFOCUS-THAIREC (for non-mental health workers) |
| **Appropriateness**  (Perceived fit; relevance; compatibility; suitability; usefulness; practicability) | - Lived experience of mental health condition or recovery journey should be brought to the training through appropriate selection of trainers. - Lived experience trainers such as peer support workers, village health volunteers, carers should be well prepared before support delivering the training | - Trainers: should include professionals, peer support workers, village health volunteers, staff who have lived experience with mental health condition, and carers - Offer peer support workers, village health volunteers, and carers additional skills before joining as a trainer e.g., communication skills |
|  | - Trainees should include nonprofessional staff (peer support workers and village health volunteers) as they are directly and closely involved with service user care within community. - Consider the needs of different types of trainees when organising and scheduling training | - Trainees: should include mental health and non-mental health staff, and non-professionals such as peer support workers and village health volunteers - Offer separate session for healthcare professionals and non-professional trainees due to the diversity of their educational background and experience |
| **Feasibility**  (Actual fit or utility; suitability for everyday use; practicability) | - Secure funding by highlighting the importance and impact of recovery training to service users | - Have gate keepers who work in related institutions to help coordinate with stakeholders and funders - Secure funding to run the training (from ministry of public health, local authority etc.) - Test effectiveness of the training through randomised control trials to secure further funding |
| **Sustainability**  (Maintenance; continuation; durability; incorporation; integration; institutionalisation; sustained use; routinisation;) | - Integrate recovery training into existing care programme or workflow - Provide accessible refresher training (e.g., online) | - Make training publicly available - Offer certificate after training - Provide online modules and refreshing training - Provide additional recovery care/practice consultation after training - Ensure that recovery outcome measures are implemented into routine practice - Select and support a recovery ‘champion’ within the team |
