# Supplementary material for: Cultural Adaptation and Implementation Strategy of a Recovery‐Oriented Mental Health Training Intervention (REFOCUS‐THAIREC) for Healthcare Workers in Thailand: An Experience‐Based Co‐Design
Source: Health Expect. 2026 Jun 22;29(3):e70738. doi: 10.1111/hex.70738 (PMC13287322; doi:10.1111/hex.70738)
Supplement: Supplementary file 4 — Supporting File 4 [file HEX-29-e70738-s003.docx]

**Appendix 4: Supporting quotes across adaptation and implementation strategy categories**

| **Priority** | **Categories** | **Quotes** |
| --- | --- | --- |
| **Priority 1:** Improving recovery knowledge and attitudes (especially for non-mental health staff) | Include non-mental health professionals and non-professional health workers | - “I think it would be possible [to include family members], and it would actually be a good thing. Because doctors [HCPs] don’t really experience things directly like family members or caregivers do. We could show them what it’s really like at home, it’s different from being with the doctors.” (Carer01) - “That should work and would probably be good for the patients. The VHVs have their own areas of responsibility, and they know the mental health patients in their communities. So, they can act as our eyes and ears. If they learn with us, it is good. They are closest to patients. If they have knowledge, even at a basic level, it helps.” (HCP03) |
|  | Simplify the content and familiarise trainees with recovery-oriented practice | - “Yes, it depends on the individuals. If they do not understand, they may think it is too difficult. It takes effort to comprehend and apply it. If we can simplify it and make it easy to understand, it will likely be much more successful. The challenge is that our trainees also need to know about mental health, which adds more workload for them.” (HCP05) |
|  | Include shared meaning of recovery in Thai context | - “So, then we have to think, the people who reached recovery, what does recovery mean for us? But we also need to define recovery together. What is our definition?” (HCP05) |
|  | Illustrate with realistic case studies | - “We may need to bring real examples, so it is clearer. For example, for me or other public health workers, when we hear the (recovery) concept, it is not clear. Like Sister XX and Sister YY might not picture it either. If we have real examples, then we can see the picture block by block, how to do it.” (HCP04) - ‘If we only use successful cases, staff may feel it is out of reach’ (HCP05). - ‘We do not need only perfect cases […] show ups and downs’ (HCP05). |
|  | Incorporating a catalyst film | - “For me, the first video was excellent. It really hit the point, and it allowed people to process and understand what actually happened in the situation. After that day, I changed the way I work. Ever since I saw that clip, I stopped doing it [speaking carelessly with patients].” (HCP03) - “Many staff have never seen patients’ environments. Without that touch, treatment becomes routine. […] They don’t think about how difficult it is for the patient to get there, or what the costs are.” (HCP05) - “Very difficult. But the video we watched last time (catalyst film) helped change attitudes. It showed another perspective. If we use that video, something touching, it could make them change themselves, be kinder to patients.” (HCP05) |
| **Priority 2:** Integrating collaborative and recovery-oriented care planning to usual care | Provide a recovery-oriented care plan template | - “In Thailand, if a patient has schizophrenia, we just list risks one to six, then evaluate. We do not see their perspective. […] But the [recovery] principle is to talk with patients and base the care plan on their problems, right?” (HCP02) - “When I go to see a patient, I gather information and write it down. But if I am not [working] there that day, how can [other] nurses help me to put it in? That could be a guideline. […] sometimes the patient starts with a physical illness but has psychiatric issues hidden underneath. Nurses might overlook it. Doctors might overlook it. But if we know a little, we can use this [template].” (HCP05) |
| **Priority 3:** Having a good quality of life | Provide problem solving and management skills | - “So in this case, a good quality of life would mean that he (patient) stops drinking, right? The community also provides support and care. From my perspective, having a good quality of life means being able to sleep well, eat well, and stay in a good mood. Those are essential aspects of quality of life.” (HCP03) - “A good quality of life for mother is having things under control.” (Carer01) - “Sometimes we think we’ve already handled the problem, we think it’s better, but then the same issue happens again. When it happens the second time, we have to find a new way, different from before. Then we start worrying again, will this second solution work? Will it be okay? Has the problem really been resolved, or will it come back the same way?” (HCP02) |
|  | Create or facilitate a supportive environment | - “If we speak frankly, deep inside their mind it will improve. From being in the dark side, where they never experienced happiness except from substances, once they travel, the brain chemicals bring happiness in another way. They can feel joy without drugs. Happiness has many forms. It is like opening their world.” (HCP05) |
| **Priority 4:** Being accepted and respected by healthcare professionals | Provide recovery language guide | - “[Recovery] it is about language. For example, using words like ‘crazy’ is already blaming them. We should avoid using such words.” (HCP01) - “I just want them [staff] to speak kindly to us. We already manage the patient ourselves. If staff speak carelessly, even without intent, the patient overhears and feels hurt.” (Carer01) |

| **Implementation strategies** | **Categories** | **Quotes** | |
| --- | --- | --- | --- |
| ***Potential mediators*** | Accessibility and simplicity of training | - “The first factor is that it should be friendly and easy to access for all ages and groups. The second is that it should not be too abstract. For example, when teaching older adults, we can use pictures or videos to make the model more engaging, rather than just showing written lectures.” (HCP03) | |
|  | Support from manager and executive team | - “I once met a lecturer […] who had developed a project for several years. During COVID, there was a programme that looked very promising, but when it was implemented in the community, it failed completely, despite the idea was good.” (HCP03) | |
|  | Limiting extra burden from recovery-oriented care | - “It does not necessarily need to be on paper or as an app. It could be a Google Sheet used by healthcare staff for care planning. For example, when staff visit patients at home, they could use a smartphone to reduce paperwork. The statistical part could later be applied as needed.” (HCP03) | |
| ***Potential moderators*** | The implementation of recovery outcome measure in practice | - “I want to have something to help with my assessment. It could be a tool, such as a measure, to guide me when assessing (mental health) patients” (HCP03) | |
| ***Implementation strategies*** | | |  |
| Adoption | Provide additional mental health skills (for non-mental health workers) | - “It becomes knowledge for providing care and conducting assessments. This comes from my own experience, as I (subdistrict hospital) was assigned by the district hospital to follow up with (mental health) patients. I would then call or message the senior staff (mental health nurse) to ask what I should ask when assessing a particular case. When visiting the community, I worry that I might not gather complete information or that I might touch on something sensitive. So I always ask them first to give me a scope of what they want. Then, when I visit the patient, I can focus on the right points and report back accurately. I do it this way every time.” (HCP05) | |
| Appropriateness | Preparing lived experience trainers | - “They should learn first. Only then can they share correctly.” (HCP05) - “It is very sensitive. We tried once but did not want to dig too deep.” (HCP02) | |
|  | Offer separate sessions for HCPs vs non-professional trainees | - “If VHVs train with staff, they may feel shy and not express themselves. If it is just their group, they may share more freely. It is like when we [nurses] train with doctors, we are afraid to say something wrong. Same content, but the words may be different.” (HCP04) | |
| Feasibility | Secure funding | - “As I have mentioned before, for us to be able to do this, it needs to be pushed through the Ministry. Once it reaches the Ministry, we must present the idea to them and show that the pilot project we have done really works well. For example, we could start here first. We could be among the facilitators to trial it, and if it proves effective, we can present that evidence to support funding applications.” (HCP03) | |
| Sustainability | Provide online modules and refresher training | - “That (online learning) would be good. We can update content and add things (to be up to date).” (HCP05) | |
